# Advances in natural killer cell immunotherapy for hematologic malignancies

**DOI:** 10.1080/15384047.2026.2658896

**Published:** 2026-04-25

**Authors:** Huijie Hu, Yan Xiong, Minxin Xia, Lixia Sheng

**Affiliations:** a School of Medicine, Ningbo University, Ningbo, Zhejiang Province, China; b Department of Hematology, The First Affiliated Hospital of Ningbo University, Ningbo, Zhejiang Province, China

**Keywords:** Cellular therapy, cell transfer therapy, hematologic tumors, immunotherapy, NK cell

## Abstract

Natural killer (NK) cells are a unique subset of cytotoxic lymphocytes within the innate immune system. They play a pivotal role in antiviral and antitumor immunity. NK cell-based adoptive immunotherapy has advanced rapidly in recent years. Innovative approaches, such as autologous/haploidentical NK cell infusion, chimeric antigen receptor NK cells, and NK cell engagers, have emerged, demonstrating promising potential in the treatment of various diseases. Currently, enhancement strategies for NK cell therapy primarily focus on two key aspects: improving expansion efficiency and persistence, with a positive effect on the therapy's potency and cytotoxic efficacy. The present review systematically introduces the functional mechanisms of NK cells and their specialized functional subsets. This review discusses the progress and optimization methods of NK cell therapy and provides an outlook on future research directions.

## Introduction

Natural killer (NK) cells are key cells in the human immune system. They are capable of directly killing tumor cells that lack major histocompatibility complex (MHC) class I molecules through “self-missing” and antibody-dependent cell-mediated cytotoxicity (ADCC). Compared to T cells, macrophages, and innate lymphoid cells (ILCs), NK cells have the advantages of safety, being ready-to-use and off-the-shelf, which also support their potential as “off-the-shelf” cell immunotherapy candidates. First, NK cells recognize tumors in an MHC-unrestricted manner. The cytokines they release primarily function to modulate immune responses, a characteristic that inherently lowers the risk of inducing graft-versus-host disease (GVHD) and cytokine release syndrome (CRS).^1^ Second, their cytotoxic function is mediated by the synergistic action of ADCC and the finely-tuned balance between activating and inhibitory signals transduced via surface receptors.^2^ Furthermore, NK cells can be sourced from diverse origins, are relatively cost-effective to manufacture, and are readily available for clinical use. Based on these attributes, NK cells represent promising candidates for “off-the-shelf” cellular immunotherapies and have demonstrated preliminary efficacy in various diseases, including controlling autoimmune diseases, viral infections, and cellular aging.^3^

Hematological malignancies such as leukemia, lymphoma, and multiple myeloma (MM) originate from hematopoietic cells and have high heterogeneity. Comprehensive treatment regimens combining traditional chemotherapy, targeted therapy, and sequential hematopoietic stem cell transplantation with the application of rituximab have effectively prolonged patient survival.^4^ However, some patients still face issues, including primary drug resistance, disease progression, and recurrence. Adverse reactions caused by treatment, such as cytokine release syndrome (CRS), further affect patient prognosis. Therefore, NK cell immunotherapy can target tumor cells and reshape the tumor immune microenvironment, providing new hope for patients with hematological malignancies.^5^

The present review systematically discusses the current applications of NK cell immunotherapy in hematological malignancies. First, it outlines the NK cell subsets, surface receptors, and cytotoxic mechanisms closely related to tumor treatment. Since cellular immunotherapy is influenced by the tumor microenvironment (TME), the special microenvironment characteristics of hematological malignancies have also been explored. Finally, the cell sources and strategies for enhancing the function of NK cell therapy are reviewed, and prospects for future research in this field are provided.

## NK cells in hematologic malignancies: mechanisms and barriers

### Mechanism of NK cell antitumor effect

#### Surface receptors and ligands

**Figure 1.**
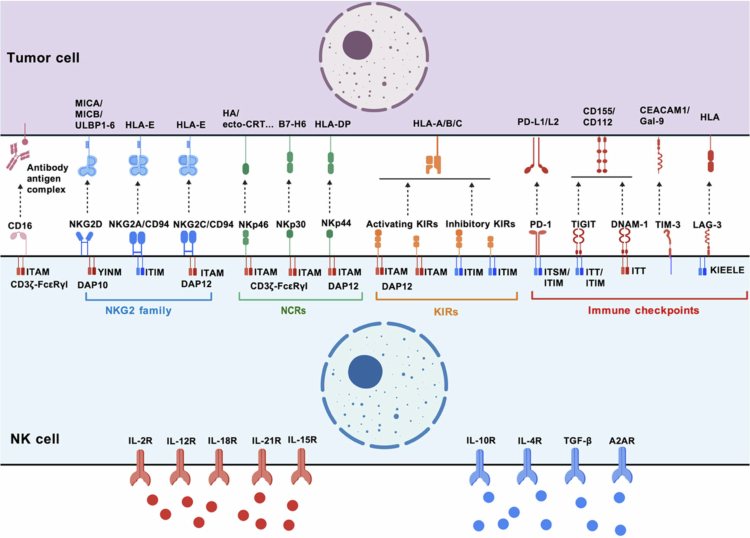
NK cell surface receptors. Natural killer (NK) cell activity is finely regulated by the balance between activating and inhibitory surface receptors. NK cell receptors can be broadly classified into five major categories based on receptor families into Fc receptors (FcRs), the NKG2 family, natural cytotoxicity receptors (NCRs), killer cell immunoglobulin-like receptors (KIRs), and various immune checkpoint receptors. In addition, NK cells express a wide range of cytokine receptors, including activating cytokine receptors, such as IL-2R, IL-12R, IL-15R, IL-18R, and IL-21R, as well as inhibitory cytokine receptors, including IL-10R and IL-4R, transforming growth factor-β (TGF-β) receptors, and adenosine A2A receptor (A2AR) (created with biogdp.com).

Unlike T cells and B cells, the balance of activating and inhibitory receptors affects the NK cells' response to target cells and also directly influences their cytotoxic activity (Figure 1).

Fc receptors (FcRs): FcR is a transmembrane glycoprotein expressed on the surface of various immune cells. It is capable of specifically recognizing the Fc segment of antibodies, thus combining their antigen recognition function with the effector function of immune cells. FcRs are divided into FcγR (ligand: IgG), FcαR (ligand: IgA), and FcεR (ligand: IgE) based on the heavy chain type of the bound antibody, regulating the functions of different immune cells.^6^ FcγR is further categorized into types I (CD64), II (CD32), and III (CD16) according to differences in affinity for IgG subclasses. FcγRIII exists in two variants, *a* and *b*, that are mainly distributed on NK cells, macrophages, and eosinophils. It is the key receptor on NK cell surfaces that can independently activate and mediate the ADCC effect. This receptor shows low affinity for free IgG1 and IgG3 and non-covalently binds to CD3ζ-FcεRγI homodimers containing immunoreceptor tyrosine-based activation motifs (ITAMs). It initiates signal cascades through ITAM tyrosine residue phosphorylation, driving NK cell cytotoxicity against tumor cells.^7^^,^^8^ The efficacy of multiple myeloma therapy is associated with CD16 expression. Notably, a low proportion of CD16^+^ NK cells^9^ within the bone marrow and a high proportion of CD16^−^ NK cells^10^ in extramedullary lesions may drive therapeutic resistance and non-response.

Killer cell immunoglobulin-like receptors (KIRs): KIRs exhibit high polymorphism due to the differences in gene composition and copy number, including six activating receptors (KIR2DS1–5, KIR3DS1), seven inhibitory receptors (KIR2DL1–3, KIR2DL5, and KIR3DL1–3), and one bifunctional receptor KIR2DL4, with their main ligands being human leukocyte antigen (HLA) class I molecules.^11^ Activating KIRs bind to DAP12 containing immunoreceptor ITAMs, recruiting spleen tyrosine kinase and zeta-chain-associated protein kinase 70 and leading to NK cell cytotoxicity via pathways, such as the Mitogen-Activated Protein Kinase (MAPK). Inhibitory KIR recognizes ligands and recruits Src Homology Region 2 domain-containing phosphatase (SHP)-1/2 through immunoreceptor tyrosine-based inhibitory motifs, blocking the cytotoxic process and preventing self-tissue damage, which achieves NK cell education and promotes its functional maturation.^12^ Studies have confirmed that inhibitory KIRs are involved in reconstituting the NK cell education function after transplantation, reducing the relapse rate of leukemia.^13^ In addition, KIR expression also affects the therapeutic effect of NK cells on hematological tumors.^14^

Natural cytotoxicity receptors (NCRs): NCRs include NKp46/NCR1/CD335, NKp44/NCR2/CD336, and NKp30/NCR3/CD337, all belonging to the immunoglobulin superfamily, which typically initiates cytotoxic functions when “missing-self occurs”. The viral surface glycoproteins are the earliest discovered NKp46 ligands that mediate NK cell killing of viruses.^15^ Endoplasmic reticulum stress and calreticulin externalization are markers of chemotherapy-induced cell death and exhaustion.^16^ NKp46 can drive the externalization of calreticulin, clear endoplasmic reticulum stress cells, and enhance NK cells' ability to recognize stress cells and senescent cells.^17^ Studies also found that NKp46-high NK cells are associated with prolonged survival in cancer patients.^18^^,^^19^

NKG2 family: the NKG2 family is a group of C-type lectin receptors expressed on NK cells. The activating receptors NKG2D and NKG2C and the inhibitory receptor NKG2A are important members of this group. The NKG2D ligands include MICA, MICB, and ULBP1-6, which initiate tyrosine phosphorylation by binding to the Grb2-Vav1 complex, the p85 subunit of phosphatidylinositol-3-kinase, and the transmembrane adaptor protein DAP-10, which mediate abnormal cell clearance.^20^ NKG2D-related cell therapy has entered the clinical research stage.^21^ NKG2C forms a dimer with CD94 to activate NK cells. Its ability to activate NK cells and recognize target cells depends on the HLA-E ligand,^22^ which is related to antigen-specific immunological memory after viral infection.^23^ The inhibitory receptor NKG2A/CD94 dimer binds to HLA-E ligands, participating in NK cell education and a balanced immune response.^24^ Recent studies have revealed an accumulation of NKG2D-downregulated NK cells in patients with acute myeloid leukemia (AML), which is associated with poor prognosis, positioning NKG2D as a potential immune checkpoint in AML.^25^^,^^26^

Other receptors: other receptors on NK cells have also received considerable attention in recent years. Programmed cell death protein (PD-1) is typically not expressed on the surface of NK cells in healthy individuals. However, its expression can be upregulated by a tumor or infection stimulus. PD-1 weakens NK cell cytotoxicity after binding to its ligands PD-L1/L2.^27^ AML, MM, and diffuse large B-cell lymphoma (DLBCL) patients show upregulated PD-1 expression in peripheral blood and bone marrow NK cells, which is associated with disease progression and poor prognosis.^28^ DNAM-1 and TIGIT form a balance receptor pair on the NK cell surfaces. Both bind to their ligands CD155/CD112, and their expression imbalance affects NK cell function, participating in the occurrence and progression of malignant tumors.^29^ In addition, T cell immunoglobulin and mucin domain 3 on NK cell surfaces are upregulated during inflammation, tumor progression, and infection.^30^^,^^31^ The expression levels of these proteins correlate with NK cell cytolytic function, disease classification, and treatment outcomes.^32^

#### Immune recognition mechanisms and killing pathways

NK cells are a key component of the innate immune system that possess multiple core functions, including immune surveillance, cytotoxicity, and immune regulation. They offer higher safety profiles and hold potential for development into universal cell therapies, demonstrating unique innate advantages in antitumor immunity.^33^

**Figure 2.**
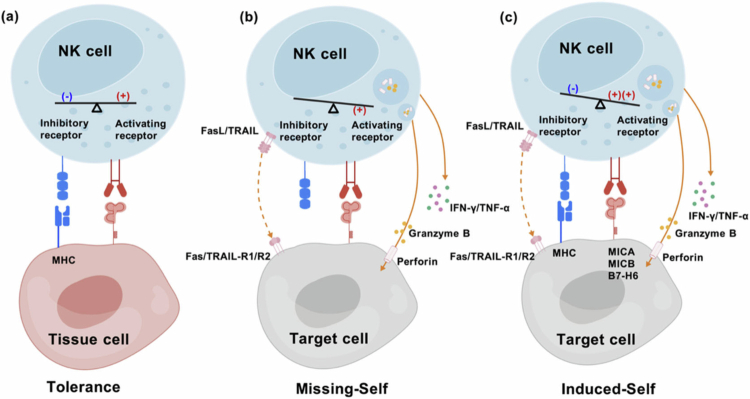
Recognition and killing mechanisms of NK cells. NK cell recognition states include three modes: self-tolerance, missing-self, and induced-self. (a) Normal tissue cells are recognized by NK cells via major histocompatibility complex (MHC)-I molecules, providing inhibitory signals to protect healthy cells from NK cell attack. (b) Tumor cells downregulate or lose MHC-I molecule expression to avoid CD8^+^ T cells, weakening the inhibitory signals from NK cells and activating them, leading to NK cell degranulation and cytotoxic activity to kill tumor cells. (c) Tumor cells or infected cells highly express activation signal ligands, stimulating NK cells to initiate antitumor/anti-infection effects (created with biogdp.com).

Missing-self: HLA, also known as MHC, comprises four functional classes: Class I genes (HLA-H, HLA-J, HLA-K, and HLA-L), pseudo-genes (MICA and MICB), classical Class I genes (HLA-A, HLA-B, and HLA-C), and non-classical class I genes (HLA-E, HLA-F, and HLA-G).^34^ HLA binds to inhibitory receptors, such as NKG2A and KIRs, thereby preventing NK cell activation and reducing damage to healthy self-cells (Figure 2). Tumor cells evade recognition by CD8^+^ cytotoxic T cells through the downregulation or loss of MHC class I molecule expression. Inhibitory receptors on the surface of NK cells detect downregulated signals when T cells fail to recognize such tumor cells, subsequently activating NK cells and initiating lysis.^35^

Induced-self: activated receptors on NK cells recognize stress-induced ligands. Several transcriptomic studies have revealed that stress events, such as infection, tumorigenesis, and DNA damage, induce activating ligand upregulation, including MICA, MICB, and B7-homolog 6 (B7-H6).^36^ Despite the normal expression of MHC class I molecules on these stressed cell surfaces, NK cells can overcome inhibitory signals on target cells by recognizing stress-related ligands via activating receptors, such as NKG2D and DNAM-1, thereby driving NK cell-mediated killing^37^ (Figure 2).

Cytotoxic pathways: NK cell-mediated ADCC effects are crucial for monoclonal antibody efficacy in treating both solid tumors and hematologic malignancies.^38^ Monoclonal antibodies, such as rituximab, specifically bind to target cell surface antigens, while their Fc fragments bind to CD16 on NK cell surfaces, activating NK cell-mediated killing.^39^ NK cells can eliminate tumor cells through three distinct mechanisms: the granzyme-perforin-mediated pathway, death receptor pathway, and cytokine-mediated immunoregulation (Figure 2). Activated NK cells form an immune synapse upon contact with target cells, undergo degranulation, and release perforin and granzymes onto the postsynaptic membrane. Perforin creates microporous channels on the target cell surface, allowing granzymes to enter the target cell through these channels. Granzymes then initiate the apoptosis-related caspase cascade reaction to exert their effect.^40^ The death receptor pathway mediates apoptosis by binding FasL and TRAIL on NK cell surfaces to Fas/TRAIL-R1/R2 on target cells, activating the death domain, recruiting Fas-Associated protein with Death Domain (FADD), and activating caspase-8.^41^ Additionally, cytokine pathways depend on NK cell secretion of cytokines, such as interferon-gamma (IFN-γ) and tumor necrosis factor-alpha (TNF-α), which can both directly inhibit target cell proliferation and promote apoptosis and enhance the antitumor assistance provided by cells, including CD8+ T cells, macrophages, and dendritic cells.^42^^,^^43^

### Cell subpopulation characteristics

NK cells can be classified into distinct subpopulations based on cell surface markers, transcriptomes, and functional characteristics (Table 1).

**Table 1.** Main NK cell subsets in humans.

Methods		Subgroup	Characteristics	Advantages	Disadvantages
Flow cytometry	Kiessling et al.^44^	cNK cell	CD56^bright^ CD16^low/^^−^	Cytokine secretion, immunomodulation, and immunosurveillance	Weak cytotoxic potential
			CD56^dim^ CD16^bright^	High cytotoxicity	Low cytokine secretion
	Lopez-Vergès et al.^45^	aNK cell	CD56^+^ CD57^+^ NKG2C^+^ KLRG1^+^	Strong cytotoxicity and cytokine secretion ability	Limited proliferation, low cytokine sensitivity, and susceptibility to exhaustion in the TME
	Dodard et al.^46^	trNK cell	Lung trNK cell: CD49a^+^ CD69^+^ CD103^+^	Rapid local immunosurveillance, tissue repair promotion, and robust metabolic fitness	Limited trafficking, weak cytotoxicity, and immunosuppressive properties
Transcription sequencing	Rebuffet et al.^47^	NK1	CXCR4^+^ JUN/JUNB^+^/CD160^+^ IFITM1^+^/PRF1^+^ NKG7^+^	Strong trafficking, cytotoxicity, and rapid activation capacity	Reduced proliferative potential and limited lifespan
		NKint	KLRC1(NKG2A)^+^ KIR^low/^^−^	Tolerance maintenance, homing potential, and robust adaptability	Limited killing capacity and susceptibility to TME
		NK2	CD16^low/^^−^ CD57^low/^^−^ CD56^+^ CD27^+^ CD44^+^ NKG2D^+^ NKp46^+^ GZMK^+^ CD122^+^ IL18R^+^	High cytokine secretion, efficient migration and homing, contributing to tissue repair	Weak cytotoxicity and susceptibility to TME suppression
		NK3	CD16^+^ CD57^+^ CD271^+^ CD2^+^ CD18^+^ CD49d^+^ KIR^+^ PRDM1^+^ KLRC2^+^ ZBTB38^+^ CCL5^+^ GZMH^+^	Strong cytotoxicity, memory-like properties, and tissue-resident capacity	Overactivation, sensitivity to external stimuli, and susceptibility to TME
	Foltz et al.^48^	eML-1	CD117^−^ CD57^−^ NKG2A^⁺^ CD39^⁺^ BATF3^+^ TOX2^+^ IRF7^+^	Enhanced cytotoxicity, cytokine production, persistence, and hypermetabolic potential	High inhibitory receptor expression and suboptimal maturity
		eML-2	PRDM1^+^ LAG3^+^ KIR3DL1^+^	Stable phenotype and enhanced specific recognition	Deficient cytokine secretion and cytotoxicity, with limited rapid response capacity

#### Surface markers

Traditionally, NK cells are divided into two subpopulations based on the differences in NK cell expression levels of CD56 (NCAM1) and CD16 (FCGR3A): CD56^bright^ CD16^low/^^−^ and CD56^dim^ CD16bright.^44^^,^^49^ CD56^bright^ CD16^low/^^−^ NK cells secrete various cytokines (e.g., IFN-γ and IL-5) to participate in immune function regulation, while CD56^dim^ CD16^bright^ NK cells secrete cytotoxic granules, including perforin and granzymes, to mediate continuous killing of infected or tumor cells. Similarly, γδ T cells that also secrete cytotoxic granules have significantly lower continuous killing effects than CD56^dim^ CD16^bright^ NK cells due to their low co-expression of granzymes and perforin.^50^^,^^51^ Adaptive NK cells with memory function are often induced after infection. They persist long-term in circulation and are typically characterized by high expression of NKG2C, KLRG1, and α4β7.^45^^,^^52^ In addition, there are unique NK cells in different tissues, also known as tissue-resident NK (trNK) cells, like lung trNK cells that express CD49a, CD69, and CD103, which have significantly higher glycolytic capacity and dependence on glucose compared to their corresponding peripheral blood NK cells.^53^ Liver trNK cells are more sensitive to lactate metabolism.^46^

#### Transcription features

Recent technologies like single-cell RNA sequencing have revealed the diverse subpopulation composition and functions of NK cells. Holmes et al. combined flow cytometry with ATAC-seq technology to propose dividing NK cells into four subpopulations as follows: CD56^bright^, CD56^dim^ NKG2A^+^, CD56^dim^ NKG2A^−^, and CD56^dim^ CD57^+^ NKG2C^high^/CD7^low^ adaptive NK cells. They also suggested that adaptive NK cells predominantly express traditional T cell cytokines, such as BCL11B, thus exhibiting memory-like characteristics.^54^ ATAC-seq combined with CITE-seq analysis identified two cell populations after IL-12/15/18 stimulation, including memory functional eML and effector functional effc NK cells. eML NK cells were further subdivided based on their CD56 expression into eML-1, which have sustained transcriptional potential, and eML-2, which show terminal differentiation trends.^48^ In addition, another study identified three distinct NK cell subsets in the blood of healthy individuals using single-cell RNA sequencing.^47^ The NK1 cluster is the most abundant in the blood, corresponding to effector NK cells, and exhibits a high-toxicity-hypermetabolic feature. The NK2 cluster is in an intermediate transitional stage and includes CD56^bright^ and early-stage CD56^dim^ NK cells. The NK3 cluster belongs to highly mature NK cells with strong cytotoxic and memory-like functions.

### NK cell dysfunction in the hematologic tumor microenvironment

Hematopoietic stem cell homeostasis, also known as the hematopoietic stem cell niche, is maintained collaboratively by endothelial cells, non-hematopoietic stromal cells, and factors, such as chemokine C–X–C motif ligand 12 (CXCL12) and thrombopoietin (TPO), in the bone marrow microenvironment.^55^^,^^56^ The hematological malignant TME niche is disrupted, exhibiting inflammatory, hypoxic, and hypermetabolic states. For example, TET3 is overexpressed in AML patients and participates in glycolysis to promote AML occurrence and development.^57^ DLBCL patients have dysregulated MYC and HIF-1α expression, leading to an inflammatory response, enhanced glycolysis, and lactate production.^58^^,^^59^ MYC, GPT2, and SLC1A5 are key molecules in the glutamine metabolism pathway that are regulated in an m^6^A-dependent manner to promote AML development and leukemia stem cell self-renewal.^60^

#### Surface receptor expression

Recent large-scale omics studies have demonstrated that the TME of hematological malignancies often presents as an immune-excluded or immune-desert type that is characterized by insufficient immune cell infiltration, persistent inhibitory receptor expression, and significant activating receptor downregulation.^61^ The main mechanisms inducing these receptor changes include inhibitory cytokine secretion and high inhibitory ligand expression on the tumor cell surface. For example, the expression of pro-inflammatory factors, such as the alarm S100, CXCL8, interferon-stimulated gene, and IFN-γ, is upregulated in the bone marrow microenvironment of AML patients.^62^^,^^63^ MM patients produce CXCR1/CXCR2 ligands in the bone marrow microenvironment, inducing the expansion of tolerant immune cells, including GZMK^+^ CX3CR1^−^ CD56^bright^ NK cells, interferon (IFN)-responsive effector T cells, and CD8^+^ T_scm_ cells.^64^ The levels of inflammatory factor secretion and the degree of immune cell suppression both affect disease prognosis.

#### Cell subpopulation changes

Compared to NK cells from healthy individuals, various NK cell subsets in the tumor immune microenvironment often show signs of imbalance in their proportions. The analysis of the NK2 cluster proportion in cancer patients showed that it indicates a dysfunction in the immunoregulatory function of NK cells in the TME.^47^ Tang et al. conducted a single-cell pan-cancer evaluation on NK cells from patients with 24 types of tumors, including lung cancer, liver cancer, breast cancer, and melanoma.^65^ They found that CD56^bright^ CD16^low/^^−^ NK cells contained five functionally heterogeneous subpopulations, among which the c2-IL-7R-RGS1 subpopulation had progenitor cell features, the c3-CCL3 subpopulation participated in immune regulation by secreting CCL3/CCL4, and the c5- cAMP-Response Element Modulator (CREM) subpopulation mediated dendritic cell recruitment via XCL1/XCL2. CD56^dim^ CD16^bright^ NK cells were further divided into nine subpopulations, comprising functionally impaired c6-DNAJB1, highly cytotoxic c7-NR4A3, and c8-KLRC2 adaptive NK cells with memory-like characteristics. The spatial distribution and functional status of these subpopulations significantly influence the tumor immune microenvironment. Ferron et al. combined transcriptomics with flow cytometry to classify NK cells into three groups: NKG2A^+^ KIR^−^ CD57^−^ (NK2A^+^), NKG2A^+^ KIR2DL^+^ CD57^−^ (NK2A^+^ KIR^+^), and NKG2A^−^ KIR^−^ CD57^−^ (NK neg).^66^ Among them, the NK2A^+^ subset highly expresses NKG2A and NKp46 and can release cytotoxic granules, showing highly effective killing activity against lymphoid leukemia cells, while the NK2A^+^KIR^+^ subset highly expresses the DNAM-1 receptor, making it more effective in killing myeloid leukemia cells. In addition, studies using single-cell transcriptome sequencing have found that NK cells in chronic lymphocytic leukemia, in addition to the classical CD56^bright^ and CD56^dim^ NK cells, include a cluster of chronic lymphocytic leukemia NK cells that highly express GNLY and FCGR3A but lack the expression of KLRG1, NCAM1, or SELL. These cells directly or indirectly affect NK cell cytotoxic function and may also influence CD8^+^ T cells.^67^

## Strategic approaches to NK cell-based tumor therapy

### *In vivo* NK cell sensitization strategies

*In vivo* sensitization employs cytokines, small-molecule sensitizers, or NK cell engagers (NKCEs) to directly activate endogenous NK cells or counteract their dysfunction. Given that the effectiveness of *in vivo* sensitization strategies relies heavily on the patient's pre-existing NK cell functionality, pre-therapeutic assessment of the NK cell status is of critical importance. However, these interventions are subject to inherent limitations, including safety risks, poor tolerability, and therapy-induced functional exhaustion. For instance, excessive cytokine stimulation can precipitate vascular leak syndrome and subsequent NK cell functional attrition;^68^ Small-molecule agents may be subject to efflux due to the activation of drug transporters *in vivo;*^69^ and the therapeutic window of NKCEs is constrained by target antigen availability, off-tumor toxicities, and the poor survival of engaged effector cells within the immunosuppressive tumor microenvironment.^70^

#### Cytokines

Cytokines provide critical signals for NK cell survival, proliferation, and functional maturation^71^ and increase chromatin accessibility, drive epigenetic remodeling, and upregulate the expression of genes related to memory-like, cytotoxic, and immunomodulatory functions.^48^ NK cell expansion and activation were enhanced in patients with recurrent AML following transplantation after treatment with an IL-2 fusion protein targeting the extracellular matrix, especially that of CD16^+^ NK cells.^72^ IL-15 complex ALT-803^73^ and super agonist complex N-803^74^ can also induce NK cell activation, proliferation, and expansion in relapsed/refractory hematologic malignancies. In addition, 84 AML patients who received a histamine combined with an IL-2 regimen in a phase IV clinical study showed an increase in the number of CD56^+^ CD16^+^ NK cells in their blood and in the expression of the activating receptors NKp30 and NKp46^75^ (Table 2). Preclinical studies further support the potential of cytokine-based strategies. For instance, treatment with NKTR-255, a polymer-conjugated human IL-15, has been shown to activate IL-15 signaling, upregulate activating receptor expression, and enhance NK cell cytotoxic activity in models of multiple myeloma.^76^

**Table 2.** Clinical progress of NK cell therapy.

Strategy	Type	Treatment methods	Malignant tumor	Clinical phase (recruiting status)	Clinical trial
*In vivo*	Cytokine	ALT-803 (IL-15)	Relapse of hematologic malignancies more than 60 d after transplantation	I (Completed)	NCT01885897
		*N* -803 (IL-15)	Maintenance therapy after hematologic tumor transplantation	II (Recruiting)	NCT02989844
		*N*-803 (IL-15)	Relapsed/refractory indolent non-Hodgkin lymphoma	I/II (Terminated)	NCT02384954
	NKCEs	AFM-13 (CD30/CD16a)	Relapsed/refractory Hodgkin lymphoma	II (Completed)	NCT02321592
		AFM-13 (CD30/CD16a)	Relapsed or refractory peripheral T cell lymphoma	II (Terminated)	NCT05883449
		GTB-3550 (CD16/IL-15/CD33)	High-risk myelodysplastic syndrome, refractory/recurrent AML	I (Recruiting)	NCT03214666
		SAR443579 (CD123/NKp46/CD16a)	Relapsed or refractory acute myeloid leukemia, B-cell acute lymphoblastic leukemia, high-risk myelodysplastic syndromes	1/II (Terminated)	NCT05086315
Adoptive NK cell therapy	PB-NK cells	FATE-NK100	Acute myeloid leukemia	I (Completed)	NCT03081780
	UCB-NK cells	AB-101	Relapsed/refractory Hodgkin lymphoma, CD30-positive peripheral T cell lymphoma	II (Terminated)	NCT05883449
	iPSC-NK cells	FT516	CD20-positive B-cell lymphoma	I (Terminated)	NCT04023071
	NK cell lines-derived NK cells	activated natural killer cells	Resistant/relapsed acute myeloid leukemia	I (Completed)	NCT00900809
	CAR-NK Cells	CD19 iPSC-CAR-NK	CD19-positive lymphoma	I/II (Completed)	NCT03056339
		FT596	Relapsed or refractory B-cell lymphoma	I (Recruiting)	NCT04245722

Due to the large number of experiments in each category, representative experiments were selected to illustrate the research mentioned in this review. NKCEs: NK cell engagers; PB: peripheral blood; UCB: umbilical cord blood; iPSC: induced pluripotent stem cells; CAR: chimeric antigen receptor; IL-15: interleukin-15.

#### Small-molecule sensitizers

Combining small-molecule sensitizers can reprogram tumor cell phenotypes, enhance the function and metabolic adaptability of NK cells, providing new strategies to improve the efficacy of NK cell immunotherapy. Niacinamide-expanded NK cells can stably induce CD62L, guide NK cell homing, and exhibit protective metabolic features like antioxidant stress.^77^ In this clinical study, 19 patients with advanced non-Hodgkin lymphoma treated with such nicotinamide-expanded NK cells achieved an overall response rate of 74%. Antibody‒drug conjugates (ADCs), which combine cytotoxic agents with antibody fragments, offer another avenue to enhance NK cell cytotoxicity.^78^ The BCMA-targeting ADC belantamab mafodotin, in combination with dexamethasone and bortezomib, yielded an overall survival rate of 84% in patients with relapsed/refractory multiple myeloma.^79^ In parallel with these clinical advances, multiple preclinical strategies are under active investigation. For example, nicotinamide riboside and *Magnolia officinalis* extract can restore NK cell cytotoxicity and enhance their anti-leukemia activity by reducing cellular lysine lactylation levels.^80^ And the proteasome inhibitors can alter the surface proteome and enhance NK cell efficacy against leukemia.^81^ These strategies significantly enhance NK cell activity in inhibitory microenvironments by synergistically regulating signal activation, immune metabolism, and effector functions and provide new directions for further exploration of their therapeutic potential.

#### NKCEs

NKCEs utilize the characteristics of activating receptors on NK cells, forming a bridge between tumor cells and NK cell-activating receptors, thereby triggering NK cell degranulation and cytokine release and enhancing antitumor efficacy. More than 10 different NKCEs have entered clinical development stages with the rapid progress of this field (Table 2). Their molecular forms have evolved from initially simple bispecific designs to more complex and functionally potent multispecific designs. AFM-13 is a CD16A/CD30 bispecific NKCE developed by Affimed based on the redirected optimized cell killing platform. It is currently one of the more mature bispecific NKCEs. It has demonstrated good antitumor activity and tolerability in early clinical trials for relapsed/refractory Hodgkin lymphoma^82^ and has achieved significant therapeutic effects when used in combination with umbilical cord blood-derived NK (UCB-NK) cells in the treatment of patients with CD30^+^ peripheral T-cell lymphoma.^83^ CD16/CD33 tri-specific T-cell-engager GTB-3550 TriKE achieves sustained NK cell activation via an IL-15 linker, inducing a continuous and safely controllable expansion effect in patients with high-risk myelodysplastic syndromes and refractory/recurrent AML.^84^ Potential targets of NK cell-activating receptors, such as NKp30, NKp46, and NKG2D, in addition to CD16a, are gradually becoming a focus, driving the development of tri- and tetra-specific NKCEs. The triple-function NK cell binder SAR443579 simultaneously binds to NKp46, CD16a, and CD123, achieving complete remission in 33.33% of patients in phase I/II trials for relapsed/refractory AML, B-cell acute lymphoblastic leukemia, or high-risk myeloproliferative patients.^85^ In addition, a novel tetra-specific NK cell bispecific antibody NKp46/CD16a/CD20, which promotes NK cell proliferation through IL-2 mutants, reduces Regulatory T cells (Treg) activation, decreases the incidence of adverse events like pulmonary edema, and exhibits superior antitumor activity in the treatment of B-cell Non-Hodgkin Lymphoma (B-NHL) compared to rituximab and T cell bispecific antibodies.^86^

### Adoptive NK cell therapy

**Figure 3.**
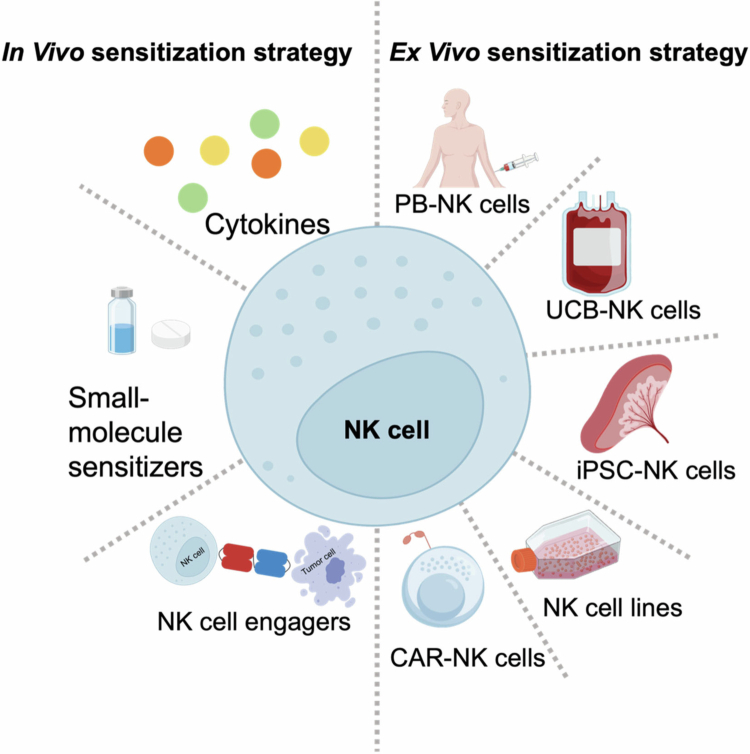
NK cell immunotherapy strategies. NK cell enhancement strategies include *in vivo* and *in vitro* enhancement approaches. *In vivo* enhancement strategies involve cytokine, small-molecule sensitizers and NK cell engagers. *Ex vivo* expansion strategies are categorized based on the cell source into NK cells derived from peripheral blood (PB), umbilical cord blood (UCB), induced pluripotent stem cell (iPSC) and NK cell lines. Additionally, *in vitro* NK cell function can be enhanced through chimeric antigen receptor (CAR)-NK cells.

Adoptive NK cell therapy is an adoptive immunotherapy strategy that involves activating and expanding NK cells from different sources *in vitro* and then reinfusing them into the patient to enhance the number and activity of NK cells in the patient's body, thereby achieving the purpose of modulating immunity and targeting tumor cells for killing (Figure 3, Tables 2 and 3).

**Table 3.** Comparison of different NK cell optimization strategies.

Strategy		Advantages	Limitations
Cytokines		Induces memory-like NK cells, and synergizes with CAR-NK/NKCEs	Toxicity risk, short-acting, and complexity
Small-molecule sensitizers		TME remodeling, synergy with other strategies, and flexible oral/injectable administration	Limited mechanism; unknown side effects
NKCEs		Bridging activation, high efficacy, broad applicability, and mature manufacturing process	Receptor-dependent, variable efficacy, and high development complexity
CAR-NK cells		Low CRS/GVHD risk and multimodal killing capacity	Low transduction efficiency and poor *in vivo* persistence
NK cells from different sources	PB-NK cells	Mature cells, widely available sources, and low alloreactivity from autologous sources	Difficult to expand, reduced post-cryopreservation viability, and challenging genetic engineering
	UCB-NK cells	Widely available, low alloreactivity, and suitable for “off-the-shelf” use	Low maturity and low receptor expression
	iPSC-NK cells	Unlimited expansion, high homogeneity, and amenable to universal donor cell line generation	Long manufacturing duration, potential epigenetic memory, and tumorigenic risk
	Cell line-derived NK cells	Unlimited expansion, high genetic engineering efficiency, low cost, no HLA matching required	Poor *in vivo* persistence and functional impairment

This table provides a comparative overview of key strategies for optimizing NK cell therapies, including cytokines, small-molecule sensitizers, NK cell engagers (NKCEs), and chimeric antigen receptor (CAR)-NK cells. We also compare NK cells derived from different sources, such as peripheral blood (PB), umbilical cord blood (UCB), Induced pluripotent stem cell (iPSC) and the NK-92 cell line.

#### Autologous and haploidentical sources

Adoptive NK cell therapy was initially focused on autologous NK cells, which have the advantage of no immune rejection and GVHD risk.^87^ However, autologous NK cells have a limited efficacy in patients with a high tumor burden due to their recognition of self-MHC-I molecules leading to tumor-induced NK exhaustion. However, they still hold significant value in low tumor burden and consolidation therapy for malignant diseases.^88^ A study has shown that infusing autologous NK cells after autologous stem cell transplantation in MM can enhance NK cell numbers and activity and reduce minimal residual disease.^89^

Haploidentical NK cells take advantage of their natural KIR-HLA mismatch, showing safety, low GVHD risk, and significant antitumor activity in the treatment of diseases, such as plasma cell myeloma^90^ and AML.^91^ Approximately 72% of children with relapsed or refractory disease achieved complete remission after haploidentical NK cell infusion.^92^ The remission rate reached 60% in elderly refractory AML patients after infusion.^93^ However, reliance on amplification technology,^94^ *in vivo* persistence,^95^ and donor-recipient KIR/HLA mismatch reducing efficacy^96^ remain key bottlenecks limiting the widespread application of adoptive NK cell therapy.

#### Alternative donor source

Peripheral blood-derived NK (PB-NK) cells. Peripheral blood is a commonly used source of NK cells, which show complete expression of killing receptors and differentiation maturity. They can continuously expand *in vivo* after infusion^97^^,^^98^ and promote immune reconstitution.^99^ Phase I clinical trials showed that children with recurrent AML after transplantation achieved sustained disease remission after receiving NK cells from healthy donors.^100^ However, the proportion of NK cells in peripheral blood is not high, so they need to be extensively expanded *in vitro* to obtain sufficient numbers for treatment.

UCB-NK cells. UCB-NK cells have abundant sources and a strong proliferation ability. Their immune function regulation is superior to that of peripheral blood, manifested as more diverse immune checkpoint-related subpopulations and upregulated IFN-γ expression.^101^^,^^102^ CD16 and other activating receptors were continuously expressed in elderly AML patients after receiving NK cells from umbilical cord blood, and their residual disease turned negative.^103^ UCB-NK cell type AB-101 has also achieved a persistent complete remission when combined with rituximab in relapsed/refractory B-cell non-Hodgkin lymphoma.^104^

Induced pluripotent stem cell-derived NK (iPSC-NK) cells. iPSC-NK cells have uniform expansion characteristics. A series of preclinical studies have confirmed that iPSC-NK cells targeting MM,^105^ B-cell lymphoma,^106^^,^^107^ and acute leukemia^108^ have persistent antitumor effects. A clinical trial jointly conducted by eight major research centers in the United States indicated that patients with recurrent/refractory diffuse large B-cell lymphoma who received iPSC-NK cells FT516 achieved an objective response rate of 58%, and no dose-related toxicity or treatment-related deaths were observed.^109^ However, iPSC-NK cells carry potential risks of malignant transformation and immunogenicity.^110^ Recent studies have shown that genetic deletion of the adhesion ligands CD54 and CD58 can effectively reduce the rejection response of host immune cells, providing new insights for universal cell therapy.^111^

Cell line-derived NK cells. The NK-92 cell line is the first NK cell source approved by the Food and Drug Administration for cellular therapy. It is dependent on interleukin expansion and displays a high activation, low inhibitory receptor phenotype. Boyiadzis et al. injected NK-92 cells into seven patients with relapsed/refractory AML and found that the expression levels of IL-1β, IL-1Rα, and IL-12 significantly increased with the infusion dose.^112^ Liu et al. confirmed that NK-92 cells have a high killing efficiency against childhood T cell and precursor B-cell acute lymphoblastic leukemia by co-culturing them with HLA-E-expressing cells.^113^ However, the NK-92 cell line belongs to aneuploid cells. Owing to their lack of CD16 and NKp44 expression, these cells require irradiation before infusion, a process that also leads to a short lifespan and limited persistence of NK cells derived from the NK-92 cell line.^114^

#### Chimeric antigen receptor NK (CAR-NK) cells

CAR-NK cells introduce the CAR structure into NK cells. Compared to the limitations of chimeric antigen receptor T (CAR-T) cells, such as fratricide,^115^ CAR-NK cells combine the specificity of recognition with the functions of NK cells, thereby reducing the risks of CRS and GVHD.

The U.S. Clinical Trials Database lists 106 CAR-NK clinical trials related to the blood system. The vast majority of B-cell malignant tumors have a high expression of CD19, which is a key factor in B-cell survival and malignant transformation processes and one of the ideal targets for treating B-cell non-Hodgkin lymphoma.^116^ The first CAR-NK cell clinical trial in the United States reported that a 64% complete response (CR) rate was achieved in 11 patients with relapsed/refractory non-Hodgkin lymphoma and chronic lymphocytic leukemia after a single infusion of CD19 CAR-NK cells.^117^ Ghobadi et al. applied CAR-iPSC-NK therapy to follicular lymphoma patients, achieving an objective response rate (ORR) of 100% and a CR of 85%.^118^ Other target points, such as CD226 downregulation, are involved in AML immune escape.^119^ CD226 CAR-NK cells in patient-derived xenograft (PDX) models can reduce tumor burden and prolong patient survival.^120^ High CD123 expression is associated with poor AML prognosis. CD123 CAR-NK cell cytotoxicity is lower than that of CD123 CAR-T cells, although the efficacy is comparable.^121^ In recent years, there have been developments in the areas of dual CAR systems^122^ and armored CAR-NK cells,^123^^,^^124^ which integrate metabolic regulation, microenvironment adaptation, and combination strategies, thereby enhancing antitumor cell activity, persistence, and safety.

Adoptive NK-cell therapy has shown a good safety profile and a degree of remission rate in various hematological malignancies. However, issues such as insufficient persistence *in vivo*, inefficient transduction, off-target effects, and homing disorders, limit its application. On the one hand, NK cell products lack memory-like subsets and depend on cytokine signals.^125^ In addition, the highly immunosuppressive TME of hematological malignancies^126^ promotes their functional exhaustion and apoptosis. On the other hand, methods like viral transduction and electroporation have low transfection efficiency, affect membrane stability, and result in high cell death rates^127^ and potential malignant transformation risks,^128^ leading to high NK cell therapy costs. Additionally, the TME in hematological tumors expresses CXCL9 and other cytokines, reduces or even loses antigen expression,^129^^,^^130^ and interferes with NK cell localization and recognition, leading to homing disorders and increased risk of tissue damage.

## Clinical translational challenges and strategic opportunities

### Challenges to clinical translation

Despite the remarkable efficacy demonstrated by NK cell-based therapies in hematologic malignancies, their broader clinical translation is constrained by several critical hurdles, including poor *in vivo* persistence, defective trafficking, and off-tumor toxicity.

NK cell dysfunction within the immunosuppressive TME. The efficacy of both adoptively transferred NK cells and endogenous NK cells stimulated by agents such as cytokines or NKCEs is severely compromised within the hostile TME of hematologic cancers. Characterized by hypoxia, metabolic dysregulation, and chronic activation, the TME profoundly drives NK cell dysfunction. This is manifested by genome-wide epigenetic reprogramming,^131^ metabolic decline,^132^ and immunosuppression,^133^ collectively limiting the persistence and anti-tumor activity of these effector cells.

Defective trafficking and homing. The homing efficiency of NK cells to tumor sites, particularly the bone marrow niche, is often impaired by factors within the TME. For instance, in multiple myeloma, the upregulation of CXCR3 ligands (CXCL9, CXCL10) within the microenvironment can disrupt NK cell migration to the bone marrow,^130^ thereby diminishing their anti-tumor efficacy.

Antigen-related limitations: “On-target, off-tumor” toxicity may arise when target antigens are expressed on healthy tissues. Conversely, therapeutic efficacy can be undermined by immune evasion mechanisms, such as reduced antigen density or complete loss of antigen expression by malignant cells.^129^

Challenges in genetic modification. The development of engineered NK cell products is highly dependent on efficient gene delivery methods, including viral transduction, DNA transfection, and mRNA transfection. However, this approach faces two major obstacles. First, the intrinsic innate immune mechanisms of NK cells can render them resistant to certain delivery vectors, and the poor proliferative capacity of subsets such as CD56^dim^ NK cells further limits transduction efficiency. Second, physical transfection methods, such as electroporation, which create transient pores in the cell membrane,^127^ can increase membrane instability. Furthermore, the risk of random genomic insertions raises concerns about disrupting normal NK cell development or even inducing malignant transformation,^128^^,^^134^^,^^135^ posing significant challenges to the safety and efficacy of NK cell therapies.

Therefore, these multifaceted challenges underscore the urgent need for a systematic assessment of NK cell subset characteristics within the hematologic TME, the identification of actionable biomarkers, and the development of safer and more efficient next-generation NK cell strategies.

### Future outlook

NK cell therapy shows potential in hematological malignancies. However, a key bottleneck limiting its clinical translation is the insufficient systematic characterization of TME heterogeneity in these diseases. Future research should leverage high-resolution analytical tools—such as immuno-oncology biological research (IOBR)^136^ and TMEtyper^137^ to comprehensively deconvolute the composition of the hematologic TME. These approaches can guide the optimization of individualized immunotherapy strategies. The integration of these advanced technologies is driving a paradigm shift toward precision immuno-oncology. A central requirement for achieving this goal is the identification of specific biomarkers capable of predicting therapeutic response. Emerging evidence has revealed correlations between distinct molecular markers and immune infiltration or treatment outcomes—For instance, MITD1 with immune infiltration in hepatocellular carcinoma^138^ and the pro-inflammatory cytokine OSMR with a treatment response in acute myeloid leukemia.^139^ Given the profound complexity of the TME, multidisciplinary integration strategies, such as nanomedicine engineering^140^ and next-generation cancer vaccines,^141^ are opening new therapeutic avenues for hematologic malignancies. Collectively, the optimization of NK cell therapy is advancing along two major trajectories: (1) genetic engineering to enhance the targeting capacity, effector function, and *in vivo* persistence of NK cells and (2) the induction of memory-like NK cells to prolong their survival and potentiate antitumor efficacy.

#### Genetic engineering

The application of genetic engineering technology can help to directly optimize cells. On the one hand, precise knockout of specific genes improves cell function. CREM knockout can reshape the microenvironment and enhance the antitumor characteristics of CAR-NK cells.^142^ The Selection by essential-gene exon knock-in (SLEEK) technology can achieve over 90% knock-in efficiency in iPSC-NK cells, significantly improving the persistence and tumor-killing ability of NK cells.^143^ In addition, gene transfer technology improves transfection efficiency. Using mRNA co-transfection technology to synchronously introduce fucosyltransferase-7 and function-enhanced CXCR4 into NK cells can restore their homing and adhesion capabilities.^144^ Transposons are DNA sequences that can self-replicate and move within the genome. These genes are also known as jumping genes. Currently, Sleeping Beauty and piggyBac are the two main transposon types. NK cells derived from peripheral blood modified by Sleeping Beauty show a higher CAR expression and greater potential for targeting leukemia and lymphoma.^145^ However, the piggyBac transposon has a higher gene integration efficiency and larger gene loading capacity, allowing modified cells to stably expand and effectively inhibit tumor growth.^146^ On the other hand, gene circuits achieve precise cell regulation through “logic gate control.” Daver et al. transferred circuit-gated components into NK cells, making them to target and kill CD33^+^/FLT3^+^ AML cells and precisely release IL-15 to enhance migration ability.^147^ Bi et al. designed an intelligent NK cell complex loaded with chemotherapy drugs and IL-21, which utilizes highly reactive oxygen species characteristics in the TME and highly reductive properties after chemotherapy to enhance their homing ability and achieve synergistic effects.^148^

#### Memory-like NK cells

The poor persistence of NK cells limits their development and application, although some NK cell subsets have similar memory functions, making them applicable for enhancing NK antitumor effects.^52^ There have been reports that NK cell pre-treatment with IL-12, IL-15, and IL-18 induces their differentiation into memory-like (ML) cells, which are then infused into patients with myeloid malignancies who have relapsed after transplantation. The infusion showed good tolerance and achieved 10- to 50-fold expansion of cells.^149^ In addition, a clinical trial confirmed that ML-NK cells infused into relapsed/refractory AML patients achieved complete remission in 87% of patients while eliminating high-risk mutations.^150^

## Conclusion

NK cell immunotherapy is rapidly developing, showing great potential while facing multiple challenges, including low gene transfection efficiency, immune suppression, off-target effects, and insufficient cell persistence. Strategies such as gene editing technology, cytokine engineering, and metabolic intervention have been able to enhance NK cell proliferation, killing ability, and homing capacity in recent years. However, existing optimization strategies mainly focus on improving efficacy, lacking systematic analysis of NK cell subset heterogeneity and differences in the immune microenvironment of hematological malignancies, which constitute significant limitations.

Therefore, future research should integrate multi-omics technologies to carry out in-depth analysis of the characteristics of various NK cell subsets in the immune microenvironment, construct “intelligent” engineered NK cells through immune‒metabolic co-regulation, and develop more effective treatment strategies. These precisely engineered designs will significantly broaden the therapeutic window of NK cell therapy, providing safer and more extensive clinical application prospects for addressing tumor heterogeneity and recurrence.

## Data Availability

No data was used for the research described in the article.
